# The cardiac distress inventory: A new measure of psychosocial distress associated with an acute cardiac event

**DOI:** 10.1186/s12872-022-02897-y

**Published:** 2022-11-03

**Authors:** Alun C Jackson, Michael R Le Grande, Michelle C Rogerson, Chantal F Ski, John Amerena, Julian A Smith, Valerie Hoover, Marlies E Alvarenga, Rosemary O Higgins, David R Thompson, Barbara M Murphy

**Affiliations:** 1 Australian Centre for Heart Health, Melbourne, VIC Australia; 2 Centre on Behavioral Health, University of Hong Kong, Hong Kong SAR, China; 3 Melbourne Centre for Behaviour Change, School of Psychological Sciences, University of Melbourne, Melbourne, VIC Australia; 4 Integrated Care Academy, University of Suffolk, Ipswich, UK; 5 Department of Psychiatry, University of Melbourne, Melbourne, VIC Australia; 6 Barwon Health, Geelong, VIC Australia; 7 Deakin School of Medicine, University Hospital Geelong, Geelong, VIC Australia; 8 Department of Cardiothoracic Surgery, Monash Health, Clayton, VIC Australia; 9 Department of Surgery, School of Clinical Sciences at Monash Health), Monash University, Clayton, VIC Australia; 10 Division of Cardiovascular Medicine, Stanford University School of Medicine, Stanford, CA USA; 11 Institute of Health and Wellbeing, Federation University, Berwick, VIC Australia; 12 Victorian Heart Institute, Clayton, VIC Australia; 13 Monash Health & Department of Medicine, Monash University, Clayton, VIC Australia; 14 Department of Physiotherapy, University of Melbourne, Parkville, VIC Australia; 15 School of Nursing and Midwifery, Queen’s University Belfast, Belfast, UK; 16 Melbourne School of Psychological Sciences, University of Melbourne, Parkville, VIC Australia

**Keywords:** Psychological distress, Cardiovascular disease, Mental health, Scale development, Measurement, Psychometric analysis

## Abstract

**Background:**

Many challenges are posed by the experience of a heart attack or heart surgery which can be characterised as ‘cardiac distress’. It spans multiple psychosocial domains incorporating patients’ responses to physical, affective, cognitive, behavioural and social symptoms and experiences related to their cardiac event and their recovery. Although some measures of the psychological and emotional impacts of a cardiac event exist, none provides a comprehensive assessment of cardiac distress. To address this gap, the study aimed to develop a Cardiac Distress Inventory (CDI) using best practice in instrument design.

**Method:**

An item pool was generated through analysis of cognate measures, mostly in relation to other health conditions and through focus group and individual review by a multidisciplinary development team, cardiac patients, and end-users including cardiac rehabilitation co-ordinators. The resulting 144 items were reduced through further reviews to 74 for testing. The testing was carried out with 405 people recruited from three hospitals, through social media and by direct enrolment on the study website. A two-stage psychometric evaluation of the 74 items used exploratory factor analysis to extract the factors followed by Rasch analysis to confirm dimensionality within factors.

**Results:**

Psychometric analysis resulted in the identification of 55 items comprising eight subscales, to form the CDI. The subscales assess fear and uncertainty, disconnection and hopelessness, changes to roles and relationships, overwhelm and depletion, cognitive challenges, physical challenges, health system challenges, and death concerns. Validation against the Kessler 6 supports the criterion validity of the CDI.

**Conclusion:**

The CDI reflects a nuanced understanding of cardiac distress and should prove to be a useful clinical assessment tool, as well as a research instrument. Individual subscales or the complete CDI could be used to assess or monitor specific areas of distress in clinical practice. Development of a short form screening version for use in primary care, cardiac rehabilitation and counselling services is warranted.

**Supplementary Information:**

The online version contains supplementary material available at 10.1186/s12872-022-02897-y.

## Background

Both anxiety and depression are common after a heart attack and heart surgery [1–4]. In addition, acute cardiac events are often experienced as traumatic, with people having to confront their mortality for the first time [5]. Survivors of acute events have to cope with having less control over their lives than they are used to. As Vilchinsky notes, these survivors are exposed to an ongoing internal somatic threat which is not exclusively related to past experience; ‘’rather, it is chronic, and in many ways it is anchored in fears and worries about the future, vis-a-vis treatment, illness progression, potential recurrence and even death’’ [6]. The range of emotions experienced post-event can include feelings of uncertainty [7–9] and fear of progression or recurrence of the condition [10–12], leading to feelings of vulnerability [13], hopelessness [5, 13], anger and resentment [5], along with sadness and grief for the life that they envisaged now being lost or changed, and loss of usual roles and responsibilities [5, 9].

The cardiac event often challenges people’s usual ways of coping. Coping with pain and having to pay more attention to what is happening physically [5] demands new coping skills. For some, the trauma associated with the cardiac event can trigger a resurfacing of past traumas or unresolved grief, with concomitant intrusive thoughts, nightmares [5] and sleep disorders such as insomnia [14].

We have argued elsewhere for the need for a nuanced understanding of the many challenging emotions, changes and experiences that follow an acute cardiac event which we believe can be conceptualised as ‘cardiac distress’. We have defined cardiac distress as ‘’*a persistent negative emotional state rather than a transient state; involving multiple psychosocial domains; that challenges a patient’s capacity to cope with living with their heart condition, the treatment of the condition, and the resultant changes to daily living; and challenges the person’s sense of self and future orientation’’* [15, 16]. Cardiac distress spans multiple psychosocial domains, thereby incorporating patients’ responses to physical, affective, cognitive, behavioural and social symptoms and experiences related to their cardiac event and their recovery [15]. Although there are some measures of the psychological and emotional impacts of a cardiac event, none of the existing measures provides a comprehensive or detailed assessment of cardiac distress, largely due to a failure to adequately conceptualise cardiac distress [16]. Moreover, no currently existing measure enables a mental health professional to identify priority areas clearly enough to offer timely tailored psychosocial intervention for a distressed patient [17, 18].

To fill this gap in psychocardiology practice, this study aimed to develop a comprehensive measure of cardiac-related distress, using best practice instrument design, as described in a previously published paper [16]. The inventory development process described here builds on our earlier investigation of the prevalence and predictors of cardiac distress which relied on clinical knowledge to categorise items on an a priori basis [19]. The present process uses both clinical and empirical evidence to develop the new instrument, which is to be known as the Cardiac Distress Inventory (CDI).

The following description of the method adopted for creating the CDI is divided into two phases: item generation and item testing. The Results section provides full details on the outcome of application of the methods described.

## Method

### Phase 1: item generation

There were several steps in the item generation process:

i. Initial generation of items by a multidisciplinary group of researchers and clinicians representing the disciplines of nursing, psychiatry, behavioural health, psychology, and cardiology.
ii. Review of generic and condition-specific measures of distress to identify the elements comprising the construct of ‘distress’ as previously defined by the research team, in those measures and to identify items which could be used as is or adapted for the CDI.
iii. Review of cardiac-specific measures incorporating elements of distress as defined by the present authors [15]. Review of items for appropriateness for a post-cardiac event population by the multidisciplinary investigator group.
iv. Focus group testing with cardiac rehabilitation (CR) professionals from multiple disciplines.
v. Consultation with, and feedback from, cardiac patients (key informants) on the structure and content of the CDI.

### Phase 2: item testing

#### Study population

#### Inclusion and exclusion criteria

Eligible participants were those who had had an acute coronary event, namely acute myocardial infarction (AMI), percutaneous coronary intervention (PCI), coronary artery bypass graft surgery (CABGS), valve issues, heart rhythm disturbance, spontaneous coronary artery dissection (SCAD) or cardiac arrest in the previous 12 months. Patients who did not have adequate English language proficiency to read and understand the Patient Information and Consent Form (PICF) and questionnaire were excluded. The PICF and questionnaire were aimed at an 8th grade reading level.

#### Participant recruitment

Participants were recruited using three separate recruitment strategies.

- Australian hospital patients. Participants were recruited from two hospitals in Australia, one in metropolitan Melbourne (Monash Health) and one in regional Victoria (Barwon Health, Geelong).

At Barwon Health, most participants were recruited while they were inpatients at the University Hospital Geelong following their hospital admission for either AMI, CABG or PCI. At this contact, the Research Nurse provided eligible patients with a brief study outline and sought interest in participation. Interested patients were provided with the PICF; consent was obtained to re-contact participants via telephone 6 weeks later to complete the questionnaire. A small number of participants were recruited during their attendance at CR at Barwon Health.

At Monash Health, participants were recruited during their appointment at the Cardiothoracic Preadmission Clinic, prior to hospital admission for CABG, or in the Cardiac Care Unit (CCU) for those with AMI, PCI and other cardiac conditions. At this contact, the Research Nurse provided eligible patients with a brief study outline and sought interest in participation. Interested patients were provided with the PICF; consent was obtained to re-contact participants at their routine 6-8-week follow-up appointment.

The recruitment procedure changed part way through the study due to COVID-19 lockdowns which prevented patients from attending face-to-face appointments. Thus, instead of completing the questionnaire while waiting for their clinic appointments as done initially, participants were either directed to the website of the Australian Centre for Heart Health (ACHH) to use an online link to a Research Electronic Data Capture (REDCap) questionnaire or were mailed a hard copy of the questionnaire for completion at home and return in a reply-paid envelope to the ACHH.

b) US hospital patients. For the Stanford site, participants were recruited using study flyers that were posted in waiting rooms throughout the outpatient cardiology clinics at Stanford hospital. Study flyers were also offered to patients directly by cardiology providers and nursing staff in the clinics. In addition, the study flyer was posted online to the SCAD Alliance’s closed Facebook group, as well as the Hypertrophic Cardiomyopathy Association’s website.
c) Social media recruitment. Participants were also recruited using direct engagement via an international social media recruitment drive through the ACHH Facebook page. Between 13 and 2021 and 23 November 2021, four Facebook recruitment posts were boosted, resulting in 12,743 people engaging with the posts through ‘liking’, sharing or commenting on them.

The study was promoted via email through the ACHH health professional networks.

### Measures

The questionnaire took approximately 25 min to complete. No identifying information was collected as no participant follow-up was involved.

#### Demographic questionnaire:

Basic socio-demographic (age, sex, country of birth, marital status, living arrangement, employment status, educational level, close confidante, recent bereavement, financial strain, private health insurance), medical (other health conditions) and event-related information (event type, date of event, attendance at CR) was collected via self-report questionnaire, using standard questions used in previous ACHH studies.

*Cardiac Distress Inventory item pool.*: A pool of 74 items addressing various issues and concerns was generated, as outlined above. For each item, participants reported on whether or not they had experienced the issue/concern in the past 4 weeks by responding Yes or No. For endorsed items, participants then reported on the level of distress associated with the issue, using a response scale where 0 =‘no distress at all’, 1=’slight distress’, 2=’moderate distress’ and 3=’severe distress’.

*Emotion Thermometers.*: The Emotion Thermometers are single-response measures of distress (DT), anxiety (AnxT), depression, (DepT) and anger (AngT). They consist of a thermometer with numerals displayed vertically from 0 to 10. Patients rate their distress ‘over the last week’, with 0 indicating ‘no distress’ and 10 indicating ‘high distress’. A total score from all four mood thermometers (ETsum) indicates overall emotional problems. These thermometers, based on the National Comprehensive Cancer Center (NCCN) cancer distress thermometer (CDT) [20], have been shown to be a clinically sensitive measure of distress in patients with mixed cardiovascular conditions [21]. The Emotion Thermometers have good internal consistency and diagnostic capabilities in cancer patients [22] (Beck et al. 2016).

*Patient Health Questionnaire-4*:^23^ (PHQ-4): The PHQ-4 is a validated brief screener (4-items) for anxiety and depression, which combines the Patient Health Questionnaire-2 (PHQ-2) and the Generalized Anxiety Disorder-2 (GAD-2) [23]. Total scores range from 0 to 12, with 0 indicating ‘no distress’ and 12 indicating ‘severe distress’. The PHQ-4 has good reliability with pre-operative surgical patients [24] and has good prognostic value with CVD patients [25].

*Kessler Psychological Distress Scale-6*:^*26*^ (K6): The K6 is a brief measure of psychological distress which has been validated in an Australian general population [26]. The K6 is both an effective screening measure and an indicator of distress severity. Scores range from 6 to 30, with lower scores indicating higher levels of distress. The scale has excellent internal consistency reliability (α = .89 [27]) and very good discrimination between individuals with serious mental illness and without serious mental illness [28].

#### COVID-19 concern:

Participants were asked to rate their concern or anxiety about the COVID-19 situation over the past week using the same response format as the Emotion Thermometers. This question was asked to assist with ascertaining the degree to which participant-reported distress was attributable to the experience of living through the COVID-19 pandemic and associated lockdowns and other restrictions, or their cardiac condition and associated difficulties.

### Data analysis

In developing the CDI scales, methods advocated as best practices in scale development were used [29, 30]. The general approach was to extract the optimal number of scales using exploratory factor analysis (EFA), confirm dimensionality and reduce and optimize the number of items using Rasch analysis, and finally examine preliminary evidence for criterion validity using concurrent measures. Rasch analysis is often used after EFA [31–33] since it has many processes that augment factor analysis. Perhaps the main advantage of Rasch analysis is its ability to put psychological constructs such as depression or distress, on a mathematical ruler analogous to how we would measure height or weight for example [34]. Unlike EFA which is more concerned with model fit, the Rasch model accounts for the difficulty level (or extent of endorsement) of individual items and transforms responses based on ordinal scales into the ruler – an interval scale via logits [35, 36]. Importantly, the Rasch model permits analysis of spread and redundancy across a wide range of person ability (or latent construct) scores within each factor through an item-person map [35, 37]. If we rely totally on EFA factor loadings it is possible that high inter-item correlations between items due to inclusion of similar worded items may artificially contribute to high factor loadings [38], which may be at a similar location on our theoretical ruler which is not desirable because some of these items may be redundant. Unlike EFA, the Rasch approach is able to give the researcher detailed information about how participants who have high levels of the underlying latent construct (e.g. distress) endorse different items, than those who have lower levels of the construct. Finally, since a factor solution is useful only if It can be interpreted in a meaningful way [39], an iterative process that considered both the empirical data and the clinical/theoretical interpretation of scales was used throughout.

### Exploratory factor analysis

EFA was used to investigate the number of latent constructs measured by the CDI items and to match items to factors (scales). The analysis began with all 74 items with parallel analysis [40] used to inform an optimal number of factors. To avoid underfactoring (too few factors extracted) [41] we took the approach recommended by Brown (2015) [42] who stated that “factor intercorrelations above .80 or .85 may imply poor discriminant validity and suggest that a more parsimonious solution could be obtained” (p. 28). For the initial EFA, principal axis factoring (PAF) was used with a direct oblimin rotation to allow factors to be correlated. The suitability of the data for EFA was assessed using the Kaiser–Meyer–Olkin measure of sampling adequacy and the Bartlett test of sphericity. Criteria for suitability are Kaiser–Meyer–Olkin 0.8 and a *p*-value for Bartlett’s χ2 of less than 0.01. A scree plot was also inspected. Interpretation of EFA solutions involved the assessment of the meaning of the set of variables with high loadings on each factor, with loadings > 0.32 considered substantive [43]. Model fit and clinical/theoretical relevance of the factor loadings were examined using several non-orthogonal rotation techniques such as oblimin, promax and simplimax. Ultimately, a cluster-based oblique rotation method, which is thought to provide a more interpretable result than those of widely known rotation techniques [44] and provided the best model fit and clinical relevance with our data, was chosen.

Model fit was assessed by multiple tests and fit indices. Chi-square should be non-significant (*p* > 0.05), but it is not always a reliable indicator since it is sensitive to sample size and non-normally distributed data. The Standardized Root Mean Square Residual (SRMR) is also reported, as well as the Root Mean Square Error of Approximation (RMSEA), Bentler’s Comparative Fit Index (CFI), and the Tucker-Lewis Index (TLI). Hu and Bentler [45] suggest that values below 0.08 for SRMR and 0.06 for RMSEA are considered a good fit, while CFI and TLI should be 0.90 or above.

### Rasch data analysis

Rasch analysis was conducted using WINSTEPS Software Version 5.2.2 [46]. The rating scale model (RSM) in Rasch was applied to estimate the parameters [47]. The RSM assumes that the distance between thresholds of adjacent options is the same across rating scales. Separate Rasch analyses were conducted for each factor identified by EFA. Rasch analysis is an iterative process where item or person estimate cycles are repeated until essential criteria for all Rasch parameters are met. At each iteration change to model fit was checked and item deletion was rejected if model fit did not improve. In retaining or deleting items clinical and theoretical importance of items within each factor was also considered. A sample size between 250 and 500 is regarded as a good size for Rasch analysis of a well-targeted scale [48]. Thus, the current sample size of 405 in this study could provide accurate and stable person and item estimates.

Central to Rasch measurement theory is the assumption of unidimensionality which assumes that all items within a given factor (subscale) measure the same underlying latent construct. Unidimensionality was assessed using point measure correlations, item fit statistics, the Wright Unidimensionality Index, and PCA of Rasch residuals as described below.

#### Point measure correlations

Point measure correlations were examined to assess the relationship between real observations and the predicted Rasch measures. Correlations in the positive direction indicate that observations agree with the unidimensional Rasch model.

#### Principal component analysis of Rasch residuals

The principal component analysis (PCA) of Rasch residuals (PCAR) was examined to assess unidimensionality of the Rasch measure. The PCAR aims to first extract the primary (unidimensional) Rasch dimension and then to examine if the remaining residuals contribute to a meaningful secondary dimension or are random noise. Unidimensionality is considered valid when the Rasch dimension explains at least 40% variance of the observed data and the eigenvalue of the first residual contrast is not greater than 2.0. If this value is > 2.0 the first contrast of the PCAR can be further examined to identify which items load on a potential secondary dimension.

#### Item fit statistics

Fit of individual items was assessed with infit (information-weighted) and outfit (outlier sensitive) mean square statistics (optimal range: 0.5–1.5) and standardized z scores (> 2.0 representing a statistically significant misfit at 0.05 alpha level). As a general rule, it is recommended to begin fit analysis by looking at Outfit before Infit and mean-square (MNSQ) before z-standardized (ZSTD) mean. The expected value for MNSQ is approximately 1.0, and values between 0.5 and 1.5 are considered productive for measurement [37]. If the MNSQ value is beyond this range, ZSTD must be checked – ZSTD values of 2.0 or more indicate statistically significant model misfit [37].

#### Person and item reliability, and separation indices

The item and person separation indices were calculated in order to give an estimate of the spread of items or individuals along the continuum of ability (endorsement). The person reliability index represents the reproducibility of the rater observations whereas the item reliability indicates the consistency of items. The separation indices are interpreted as acceptable if values are ≥ 2.0 [49]. The reliability indices were interpreted with values ≥ 0.5 regarded as adequate, ≥ 0.80 as good, and ≥ 0.90 as high reliability [50]. An item separation index greater than 3.0 coupled with reliability greater than 0.90 is an indication that the hierarchical structure of items according to level of latent trait will be stable in a new sample [35].

#### Wright’s Unidimensionality Index

To assess how well the observed data fit the Rasch model, Wright’s Unidimensionality Index was calculated. This is the person separation index using real standard errors divided by the person separation index using model standard errors. A value of ≥ 0.9 is indicative of unidimensionality and ≤ 0.5 suggests multidimensionality [51].

#### Local independence

The assumption of local independence is met if responses to each item in within each subscale are mutually independent of the responses to another item. If there is a significant correlation among the items after accounting for the latent construct, then the items are locally dependent or there is a secondary dimension of measurement influencing the correlation [35]. Residual correlations > 0.30 were considered a concern [37].

#### Monotonicity

This assumption was tested by evaluating the rating scale for each item based on Linacre’s three essential criteria [37]. First, to ensure stability of estimates, the number of observations for each category of the rating scale was examined to confirm that there were at least 10 observations. Second, the mean ratings for each rating were examined to verify if lower ratings were associated with lower mean person ability and higher ratings with higher mean person ability. Ideally, average category measures should advance monotonically up the rating scale.

#### Differential item functioning (DIF)

DIF was used to analyze the extent to which items function differently across sub-groups such as gender or age group (split at 60 years).

### Concurrent validity and discriminant validity

The associations between CDI scale scores and well-established scales such as the Kessler (K-6) or the Emotion Thermometer were examined. Stronger significant association in Pearson correlation coefficients suggests support for concurrent validity [29]. Conversely, lower correlations between the CDI and established scales measuring different concepts (e.g., the COVID-19 Stress Thermometer) further supported discriminant validity [29].

### Discriminant accuracy in prediction of emotion Thermometer distress

Receiver operating characteristic (ROC) analysis was used to identify the optimal CDI scale cut-off score for distinguishing whether respondents experienced clinically significant distress as defined by the established cut- off thresholds for ETsum (the sum of all four mood thermometers). The area under the curve (AUC) was used to estimate the overall discriminative accuracy of the CDI scale cut- off score relative to the established cutoff scores of ETsum (a score > 14 indicates moderate and > 20 indicates severe emotional problems). Using qualitative guidelines for interpreting AUC values, namely AUC ≤ 0.70 as acceptable discrimination, AUC ≤ 0.80 as good discrimination and AUC ≤ 0.90 as excellent discrimination.

## Results

### Phase 1: item generation

**Fig. 1:**
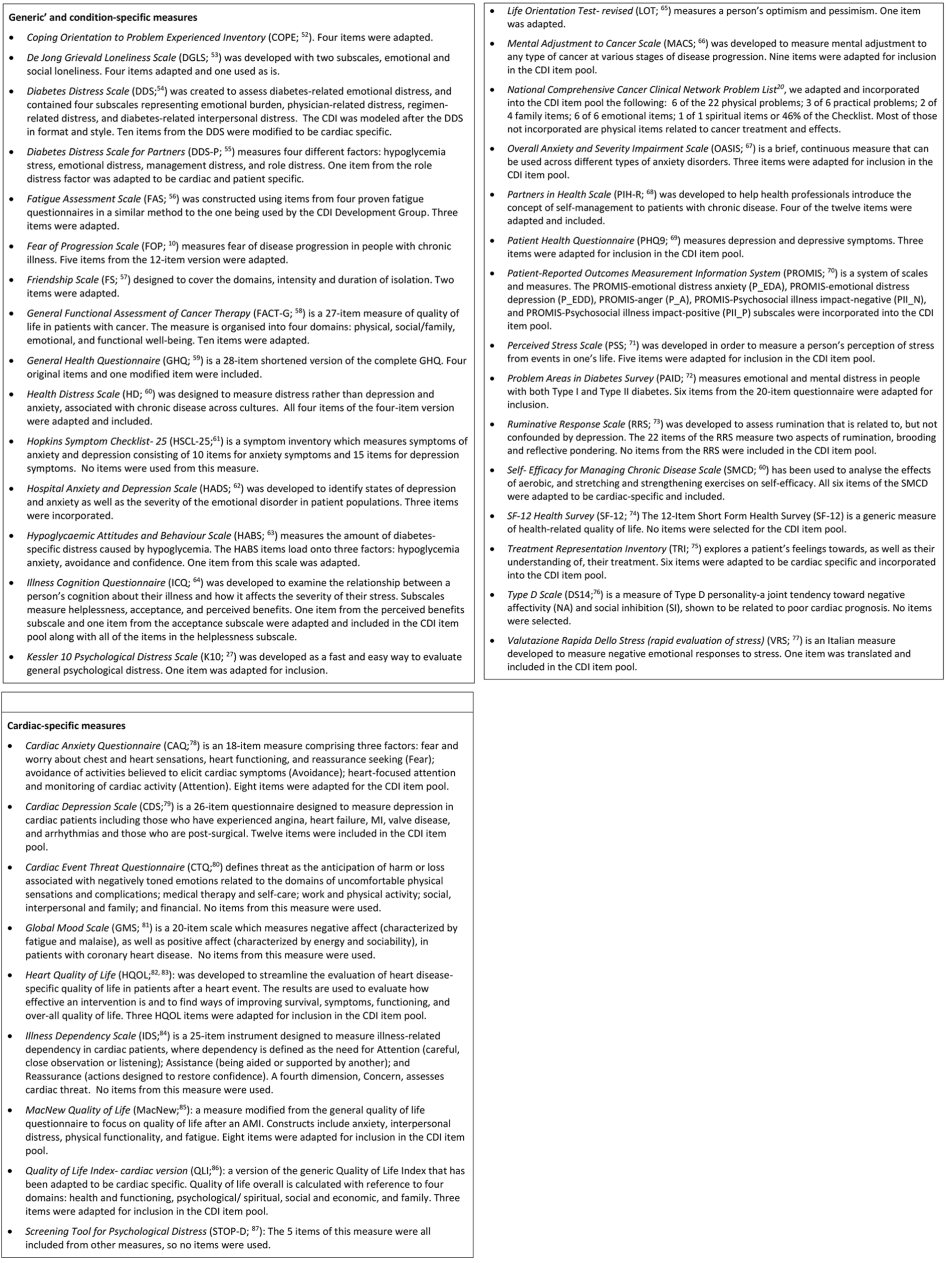
Measures reviewed for potential inclusion of items into the CDI item pool

To identify potentially relevant items from existing measures that could be adapted and incorporated into the Cardiac Distress Inventory item pool, multi database Boolean searches in EBSCO Discovery Services (include databases such as CSA Illumina, PsycInfo, PubMed, JSTOR etc.) were conducted using the search terms ‘emotional distress’, ‘psychological distress’, ‘psychosocial distress’ ‘distress measur*’, ‘cardiac’, ‘chronic illness (inc. diabetes, cancer)’. The database search was limited to English-language scholarly articles of peer-reviewed journals published from January 1990 to 2018, when this component of the study was completed. This resulted in the identification of 39 measures (generic, condition-specific and cardiac-related) used to assess elements of distress. Figure 1 provides a description of the measures and number of items used as is or adapted for inclusion in the CDI item pool.

The included items from the measures listed in Fig. 1 were reviewed by the full research team to confirm their suitability from a multi-disciplinary standpoint. Where no items were selected from a particular measure, this does not mean that there were no relevant items, but rather that at the time they were reviewed, similar relevant items may already have been selected.

Inspection of Fig. 1 shows that items from the *Cardiac Anxiety Questionnaire* and the *Cardiac Depression Scale* comprised only 15% of the total item pool. The *Diabetes Distress Scale*, on which the CDI was modelled, contributed 13.5% of the CDI test items adapted for cardiac relevance.

### Focus group testing of items

Two focus groups, comprising 27 people, were convened to test the face validity of the item pool. Focus group participants were asked to consider each of the suggested items in light of the definition of cardiac distress adopted for the study. They were asked to select those items which they believed best reflected the aspects of cardiac distress they observed in their clinical practice. The sessions were facilitated by a member of the team of investigators (AJ). One group was drawn from experienced cardiac health professionals attending an ACHH intensive course on CR (n = 14), while the other comprised members of the National Executive of the Australian Cardiovascular Health and Rehabilitation Association (ACRA) (n = 13). Two-thirds of the participants were cardiac nurses, with the remainder comprising a physiotherapist, a dietician, a psychologist, three exercise physiologists, an allied health professional, and a health promotion professional. Participants had a mean age of 41.6 years, a mean of 19.1 years of practice in their disciplines, and a mean of 9.8 years of cardiac practice. Fifty-seven per cent of the focus group participants were CR co-ordinators, seeing a mean of 128 CR patients each per annum. Item endorsement was high, and a small number of suggestions were made to add items in areas deemed not to be covered.

### Conclusion of phase 1

The processes conducted in Phase 1 resulted in a pool of 144 items that were then reviewed by the full multidisciplinary development team individually and in groups to remove duplicates, ambiguities, and to refine the item pool to reflect the conceptualisation of cardiac distress which had previously been adopted. The resulting pool was then shared with patient representatives (n = 6) from Heartbeat Victoria and Heart Support Australia. This process resulted in 74 items being selected for testing. These items were grouped into seven key conceptual domains, determined a priori by the project team, namely symptoms, self-perception, concerns about the future, negative affect, self-management, social functioning, and role functioning.

### Phase 2: item testing

## Results

Four hundred and five people were recruited for the study: 231 from hospitals and 112 from social media. A further 62 people were recruited by other means, for example, by directly enrolling from the ACHH website, or through being introduced to the study by family or friends. Characteristics of participants are presented in Table 1. Briefly, just over half the participants were male, and almost two-thirds were aged 60 and over. Approximately half were not in the paid workforce or unemployed, and around three-quarters reported some degree of financial strain. Close to a quarter reported a history of anxiety or depression. Over two-thirds had their cardiac event within the past 3 months and fewer than half had attended a CR program.

**Table 1:** Characteristics of participants

Characteristic	
Sex	
Male	216 (53%)
Female	188 (47%)
Age group (years)	
< 50	48 (12%)
50–59	109 (27%)
60–69	134 (33%)
≥ 70	112 (28%)
Education	
Primary	6 (1.5%)
Secondary	124 (31%)
Trade or TAFE qualification	103 (26%)
University diploma/degree/post-graduate	170 (42%)
Employment status	
Employee	157 (39%)
Self-employed	46 (11%)
Unemployed	25 (6.2%)
Not in the paid workforce (e.g. home duties, retired)	177 (44%)
Current financial strain	
None	109 (28%)
Slight	107 (27%)
Moderate	126 (32%)
Considerable/Extreme	48 (11.9%)
Not stated	15
Lives alone	74 (18%)
Has close confidante	334 (85%)
Marital status	
Never married	28 (6.9%)
Widowed	28 (6.9%)
Divorced or separated	60 (15%)
Married or living with partner	288 (71%)
Heart condition*	
AMI	162 (40%)
CABGS	118 (29%)
PCI	299 (74%)
HF	30 (7.4%)
SCAD	39 (9.6%)
ICD	8 (2.0%)
Comorbidities	
Obesity	61 (15%)
Diabetes	86 (21%)
Sleep Disorder	53 (13%)
Cancer	22 (5.4%)
History of Anxiety	101 (25%)
History of Depression	117 (29%)
Place of residence	
Australia	307 (75.8%)
United States of America	33 (8.1%)
Canada	13 (3.2%)
Other	52 (12.8%)
Time since cardiac event	
Less than 1 month	45 (11%)
1 to 3 months	239 (59%)
4 to 12 months	67 (16.5%)
More than 1 years	50 (12.3%)
Unknown	4
Attended cardiac rehabilitation	179 (46%)

*N* = 405. Note: Not all categories add to 405 due to missing data; * not mutually exclusive; Rasch Analysis was conducted on sub-sample with no missing data on the CDS Inventory (n-385, 95% of total sample). AMI = acute myocardial infarction, CABGS = coronary artery bypass graft surgery, PCI = percutaneous coronary intervention, HF = heart failure, SCAD = spontaneous coronary artery dissection, ICD = implantable cardioverter defibrillator

### Exploratory factor analysis

Initial EFA of the 74 CDI items revealed three items that were not loading on any factor and had questionable clinical relevance: item 62 ‘Not accepting that this has happened to me’ which could be regarded as a coping strategy (i.e. denial) rather than something people are likely to find distressing; item 61 ‘Getting lost in familiar places’ did not load onto any factor and has little clinical relevance; and item 28 ‘Having difficulty meeting my everyday expenses’ did not load highly onto any factor and conceptually did not belong in any of the factors due to it not being a consequence of cardiac distress itself.

Results of the final EFA for the seven-factor solution for the remaining 71 CDI items are shown in Supplementary Table 1. Parallel analysis resulted in 7 factors varying in size from 4 items through to 14 items. The overall Kaiser-Meyer-Olkin measure of sampling adequacy was 0.938 indicating excellent suitability of items for EFA. The 7-factor solution had good overall fit (RMSEA = 0.046 (95% CI 0.043–0.048); TLI = 0.944). The correlations between factors extracted are presented in Table 2. Correlations ranged from 0.231 to 0.64 with the majority above 0.32 and Bartlett’s test of sphericity (*χ*^*2*^ = 14888.46, *df* = 2485, *p* < 0.001) confirming the non-orthogonality of the factors. All correlation coefficients were positive thus reflecting unidimensionality and shared variance in the latent construct, cardiac distress.

**Table 2:** Pearson correlation coefficients between factors extracted in the exploratory factor analysis

	Factor 1	Factor 2	Factor 3	Factor 4	Factor 5	Factor 6	Factor 7
Factor 1	1.000						
Factor 2	0.614	1.000					
Factor 3	0.544	0.569	1.000				
Factor 4	0.520	0.500	0.641	1.000			
Factor 5	0.430	0.507	0.373	0.457	1.000		
Factor 6	0.454	0.359	0.363	0.337	0.384	1.000	
Factor 7	0.458	0.452	0.442	0.377	0.265	0.231	1.000

FACTOR 1 – Fear and uncertainty

FACTOR 2 – Disconnection and hopelessness

FACTOR 3 – Changes to roles and relationships

FACTOR 4 – Overwhelm and depletion

FACTOR 5 – Cognitive challenges

FACTOR 6 – Physical challenges

FACTOR 7 – Health system challenges

### Rasch analysis

Since all responses to items were of the same format (0,1,2,3,4), all Rasch analyses were conducted using the Rating Scale Model (RSM) in preference to the partial credit model. Seven separate Rasch RSM analyses were run for each factor identified by the EFA. Initial analyses were conducted with 0 to 4 soring including missing data. Issues with lack of monotonicity of items and disordered category thresholds were identified with these settings. Thus, analyses were re-run with cases that had missing data on the CDI removed (n = 20, leaving n = 384 for full Rasch analysis) and collapsed categories 0 and 1 (no binary endorsement and yes to binary endorsement but no level of distress) into 0 in order to run all analyses on a 0 to 3 rating score (2, 3, 4 recoded to 1,2,3). This collapse of categories affected only between 1 and 4 per cent of respondents who had indicated binary endorsement of an item but also indicated no level of distress caused. The 0 to 3 scoring system resulted in superior model fit, improved category thresholds, and no disorder of items in the Rasch model.

**Table 3:** Cardiac Distress Inventory subscales and item fit; items sorted by item endorsement within the subscale (higher to lower)

	Sub-scales	Logit measure	SE	Infit MNSQ	ZSTD	Outfit MNSQ	ZSTD
	**FACTOR 1 – Fear and uncertainty 8 items**						
1	Thinking I will never be the same again	-0.81	0.08	0.79	-3.00	0.84	-2.01
47	Not knowing what the future holds for me	-0.67	0.08	0.86	-1.90	0.81	-2.38
17	Thinking that I am not the person that I used to be	-0.53	0.08	1.02	0.27	1.09	1.04
39	Dwelling on my heart condition	-0.32	0.08	0.86	-1.92	0.84	-1.75
2	Thinking my condition might get worse	-0.02	0.08	1.09	1.09	1.14	1.29
34	Being unable to plan for the future	0.41	0.09	1.11	1.34	0.98	-0.11
36	Avoiding activities that make my heart beat faster	0.56	0.09	1.09	1.00	0.92	-0.61
30	Being in places and situations that remind me of my heart event	1.39	0.11	1.45	3.70	1.08	0.45
	Item Separation = 8.22; Item Reliability = 0.99; Person Separation = 2.00; Person Reliability = 0.80; Chronbach Alpha = 0.87						
	Wright Unidimensionality Index = 0.92						
	**FACTOR 2 – Disconnection and hopelessness 8 items**						
31	Feeling lonely	-0.63	0.08	0.89	-1.28	0.90	-0.95
55	Withdrawing from people	-0.34	0.09	0.84	-1.82	0.83	-1.52
42	Thinking my friends or family don’t understand how difficult it is living with heart disease	-0.18	0.09	0.97	-0.26	0.94	-0.46
43	Being disconnected from people in my community	-0.08	0.09	1.34	3.12	1.32	2.21
44	Being isolated from friends and family	0.03	0.09	0.98	-0.21	0.86	-0.97
46	Believing that others don’t have the same confidence in me as they did before my heart problem	0.19	0.10	0.78	-2.22	0.65	-2.58
29	Not being supported by my friends and family in my efforts to manage my heart condition	0.39	0.10	1.46	3.54	1.56	2.87
53	Being unable to accept help from others	0.62	0.11	0.99	-0.08	0.78	-1.18
	Item Separation = 3.76; Item Reliability = 0.93; Person Separation = 1.29; Person Reliability = 0.62; Chronbach Alpha = 0.86						
	Wright Unidimensionality Index = 0.90						
	**FACTOR 3 – Changes to roles and relationships 11 items**						
41	Having changes in my usual roles	-0.26	0.07	0.72	-3.79	0.76	-2.14
71	Thinking that my heart condition controls my life	-0.22	0.07	0.84	-2.03	0.82	-1.53
52	Being unable to take care of family responsibilities	-0.02	0.08	0.85	-1.76	0.82	-1.33
69	Being too dependent on others	0.33	0.08	0.9	-0.94	0.76	-1.51
65	Being unavailable to my family and friends	0.25	0.08	0.91	-0.87	0.74	-1.73
72	Becoming a burden to my family	-0.4	0.07	0.94	-0.71	0.88	-1.11
49	Not being able to go too far from home	0.02	0.08	1.03	0.4	0.98	-0.1
67	Lacking purpose or meaning in life	-0.12	0.07	1.08	0.93	0.99	-0.06
12	Not being able to return to work or continue working	-0.11	0.07	1.24	2.65	1.17	1.29
63	Being concerned about my capacity for sexual activity	-0.09	0.07	1.3	3.24	1.48	3.22
74	Thinking that my family is being overprotective of me	0.63	0.10	1.23	1.81	1.54	2.45
	Item Separation = 3.53; Item Reliability = 0.93; Person Separation = 1.35; Person Reliability = 0.65; Chronbach Alpha = 0.85						
	Wright Unidimensionality Index = 0.93						
	**FACTOR 4 – Overwhelm and depletion 7 items**						
60	Lacking energy	-0.84	0.07	1.08	0.84	1.17	1.94
51	Being emotionally exhausted	-0.21	0.08	0.76	-3.3	0.69	-3.5
26	Being irritated by little things	-0.07	0.08	0.86	-1.88	0.86	-1.4
14	Avoiding situations and activities	-0.03	0.08	0.97	-0.31	1.01	0.14
21	Being unable to deal with stress	0.19	0.08	1.09	1.13	0.93	-0.68
10	Being tearful more easily than before	0.27	0.08	1.12	1.48	1.14	1.26
48	Not being able to sustain the lifestyle changes I need to make	0.70	0.09	1.22	2.43	1.15	1.17
	Item Separation = 5.61; Item Reliability = 0.97; Person Separation = 1.85; Person Reliability = 0.77 Chronbach Alpha = 0.84;						
	Wright Unidimensionality Index = 0.97						
	**FACTOR 5 – Cognitive challenges 4 items**						
50	Forgetting things more than before	-0.81	0.1	0.99	-0.05	1.03	0.37
38	Having difficulty remembering things	-0.16	0.11	0.94	-0.71	0.91	-0.94
9	Having difficulty concentrating	0.21	0.11	0.73	-3.3	0.72	-3.12
25	Having difficulty making decisions	0.76	0.11	1.41	3.89	1.29	2.35
	Item Separation = 5.18; Item Reliability = 0.96; Person Separation = 1.37; Person Reliability = 0.65; Chronbach Alpha = 0.84						
	Wright Unidimensionality Index = 0.95						
	**FACTOR 6 – Physical challenges 8 items**						
22	Being physically restricted	-1.0	0.07	0.74	-4.12	0.74	-3.68
8	Not sleeping well	-0.73	0.07	0.98	-0.21	0.98	-0.25
6	Being short of breath	-0.44	0.07	1.15	1.99	1.27	2.95
27	Being overly aware of my heart in my chest	-0.32	0.07	0.89	-1.56	0.9	-1.13
37	Having chest discomfort	-0.17	0.07	0.79	-3.1	0.76	-2.75
24	Having bad dreams or nightmares	0.55	0.09	1.42	4.05	1.17	1.21
11	Being woken up at night by my racing heart	0.87	0.1	1.23	2.1	1.01	0.1
23	Having more pain than I can deal with	1.24	0.11	1.45	3.24	1.05	0.3
	Item Separation = 9.06; Item Reliability = 0.99; Person Separation = 1.42; Person Reliability = 0.67; Chronbach Alpha = 0.76						
	Wright Unidimensionality Index = 0.95						
	**FACTOR 7 – Health system challenges 5 items**						
66	Not getting clear directions from my health practitioner on how to manage my heart condition	-0.4	0.09	0.73	-2.88	0.68	-2.87
56	Not being able to get as much information as I want about my heart condition	-0.23	0.09	0.96	-0.32	0.91	-0.64
32	Not having access to the health care I need	0.0	0.09	1.08	0.72	0.97	-0.14
70	Not having my concerns taken seriously by my health practitioner	0.24	0.1	1.01	0.09	0.9	-0.52
45	Having difficulty getting to appointments that I need to attend	0.39	0.11	1.35	2.4	1.4	1.88
	Item Separation = 2.89; Item Reliability = 0.89; Person Separation = 0.69; Person Reliability = 0.32; Chronbach Alpha = 0.73						
	Wright Unidimensionality Index = 0.94						
	**FACTOR 8 – Death concern 4 items**						
58	Not knowing how my family will cope if something should happen to me	-0.36	0.08	1.07	0.84	1.03	0.32
3	Being afraid of dying	0.02	0.09	1.0	0.06	0.95	-0.5
19	Thinking about dying	0.14	0.09	0.9	-1.21	0.86	-1.4
68	Not knowing what will happen to other people if I die	0.2	0.09	1.03	0.33	0.93	-0.66
	Item Separation = 2.31; Item Reliability = 0.84; Person Separation = 1.28; Person Reliability = 0.62; Chronbach Alpha = 0.82						
	Wright Unidimensionality Index = 0.97						

MNSQ: Mean Square Statistic, SE: Standard Error, ZStd: Standardized Weighted/Unweighted Mean Square Fit Statistic. MnSq ideal is 1; Infit MnSq > 1.5. ZStd is only examined if MnSq indicated misfit and a ZStd > 2 indicates misfit; Wright Unidimensionality Index > 0.9 indicates unidimensionality

The initial Rasch analyses identified two problems with dimensionality (Eigen values of the first contrast in PCAR above 2.0) for Factors 1 and 3. Examination of the factor loadings identified two items in Factor 1 (item 3 Being afraid of dying and item 19 Thinking about dying) and two items in Factor 3 (item 58 Not knowing how my family will cope if something should happen to me and item 68 Not knowing what will happen to other people if I die) which affected dimensionality in both scales. The project team decided to remove these four items into a new distinct subscale (Death concern) and ran separate Rasch analyses on this subscale.

After consideration of item fit, separation of items on the Wright Map, dimensionality and DIF, the following changes to the factors identified in the EFA (Supplementary Table 1) as a result of the Rasch analyses were made: Factor 1 – drop items 18, 5, 20, 59. Move items 19 and 3 to new Factor 8 (Death concern); Factor 2 – Drop items 13, 15, 64, 33, 57; Factor 3 – Drop item 54. Move items 58 and 68 to new Factor 8; Factor 4 – Drop items 35, 4; Factor 5 – Retain all items; Factor 6 – Drop items 7, 40; Factor 7 – Drop items 73, 16; Factor 8 (new) – include items 19, 3, 58 and 68.

In total, Rasch analyses resulted in a further 16 items being dropped from the 71 items used in the EFA, resulting in a 55-item CDI consisting of 8 sub-scales (Table 3). Regarding item goodness-of-fit statistics, all item infit and outfit statistics fell within the recommended fit criteria. All items had positive point measure correlations within each scale with Wright’s Unidimensionality Index ranging from 0.90 to 0.97, thus indicating excellent unidemensionality of the latent construct within each scale. The newly constructed scale (Death concern) had acceptable item fit statistics. In addition to the Rasch statistics, all scales demonstrated high to excellent internal reliability with Cronbach alpha ranging from 0.73 to 0.87. Item-total statistics and mean scale scores for each scale are presented in Supplementary Table 2. In general, this analysis corroborated the Rasch analysis. Items that had lower fit according to the Rasch analysis also had larger potential gains to reliability if deleted. Since there were no major potential gains in reliability if an item was deleted however, we chose not to delete any items from any of the scales on this basis. The final 55 item CDI and scoring instructions is presented in the supplementary material.

### Criterion validity

**Fig. 2:**
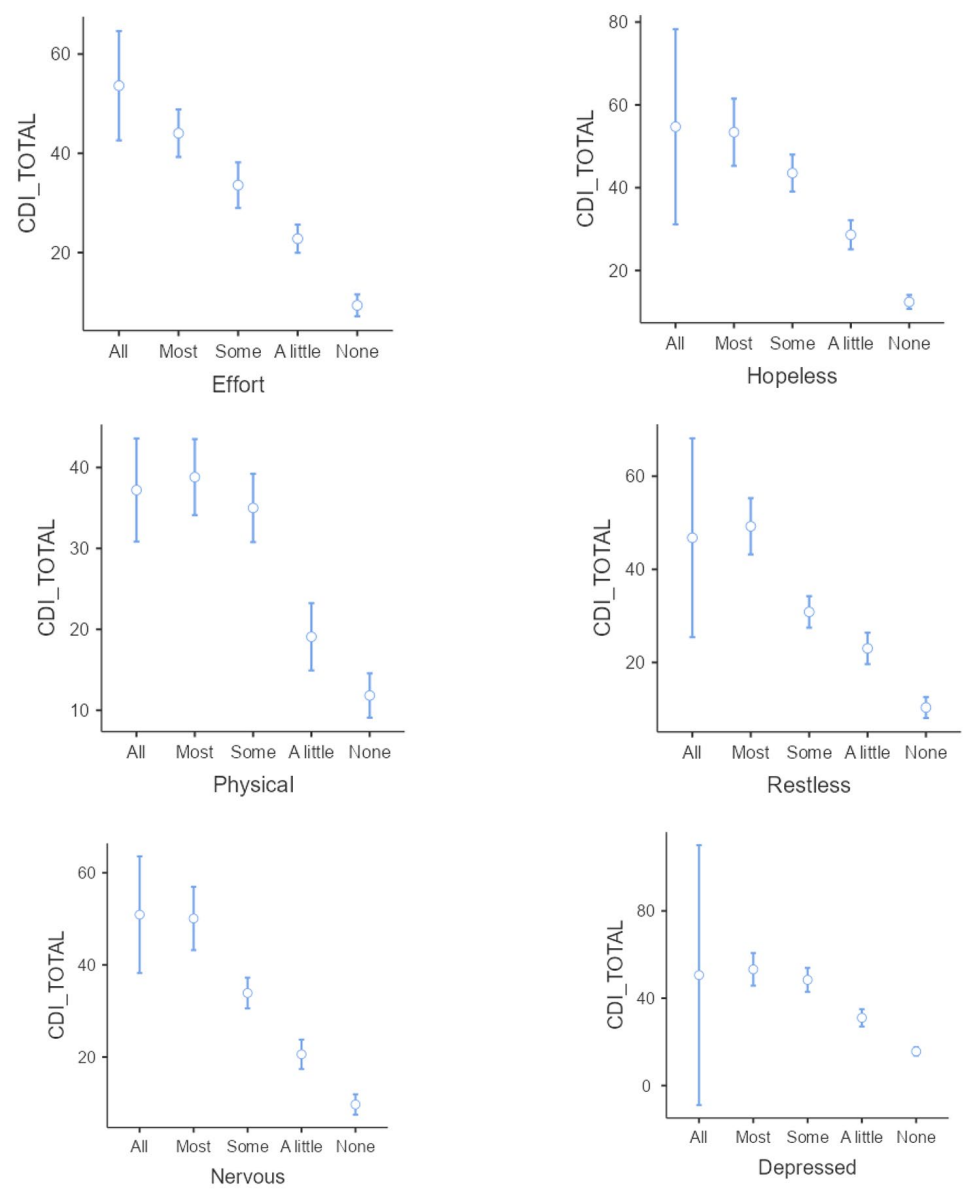
Relationship between Kessler K-6 items and CDI total score (Mean, 95% CI) Most items take the form ?In the past 4 weeks, about how often did you feel…?

Evidence of concurrent validity of the total CDI score was apparent with the highest correlation obtained with Distress Thermometer ratings and relatively lower correlations for anger and COVID-19 concerns (see Table 4). With regard to the K6, higher scores on the CDI total score were associated with higher frequencies of everything being an effort, feeling hopeless, being physically tired, being restless, nervous, and depressed (see Fig. 2). On all K6 items there were significant post-hoc comparisons between most levels of each item on mean CDI total score (e.g., for everything being an effort: all vs. a little, all vs. none, a little vs. most, some vs. none, some vs. a little, were all *p* < 0.001, a little vs. none *p* < 0.05).

**Table 4:** Pearson correlation coefficients between total CDI score and five stress thermometer ratings

		CDI_TOTAL		Anger		Anxiety		Depression		Distress		COVID-19	
**CDI_TOTAL**	Pearson’s r	—											
	*p*-value	—											
	N	—											
**Anger**	Pearson’s r	0.503	***	—									
	*p*-value	< 0.001		—									
	N	388		—									
**Anxiety**	Pearson’s r	0.692	***	0.569	***	—							
	*p*-value	< 0.001		< 0.001		—							
	N	388		387		—							
**Depression**	Pearson’s r	0.639	***	0.571	***	0.715	***	—					
	*p*-value	< 0.001		< 0.001		< 0.001		—					
	N	388		388		387		—					
**Distress**	Pearson’s r	0.700	***	0.556	***	0.787	***	0.676	***	—			
	*p*-value	< 0.001		< 0.001		< 0.001		< 0.001		—			
	N	388		387		387		387		—			
**COVID-19**	Pearson’s r	0.427	***	0.260	***	0.350	***	0.410	***	0.387	***	—	
	*p*-value	< 0.001		< 0.001		< 0.001		< 0.001		< 0.001		—	
	N	367		365		365		365		365		—	

Note. * *p* < 0.05, ** *p* < 0.01, *** *p* < 0.001

### Discriminative accuracy of the total CDI score in prediction of emotional distress

**Fig. 3:**
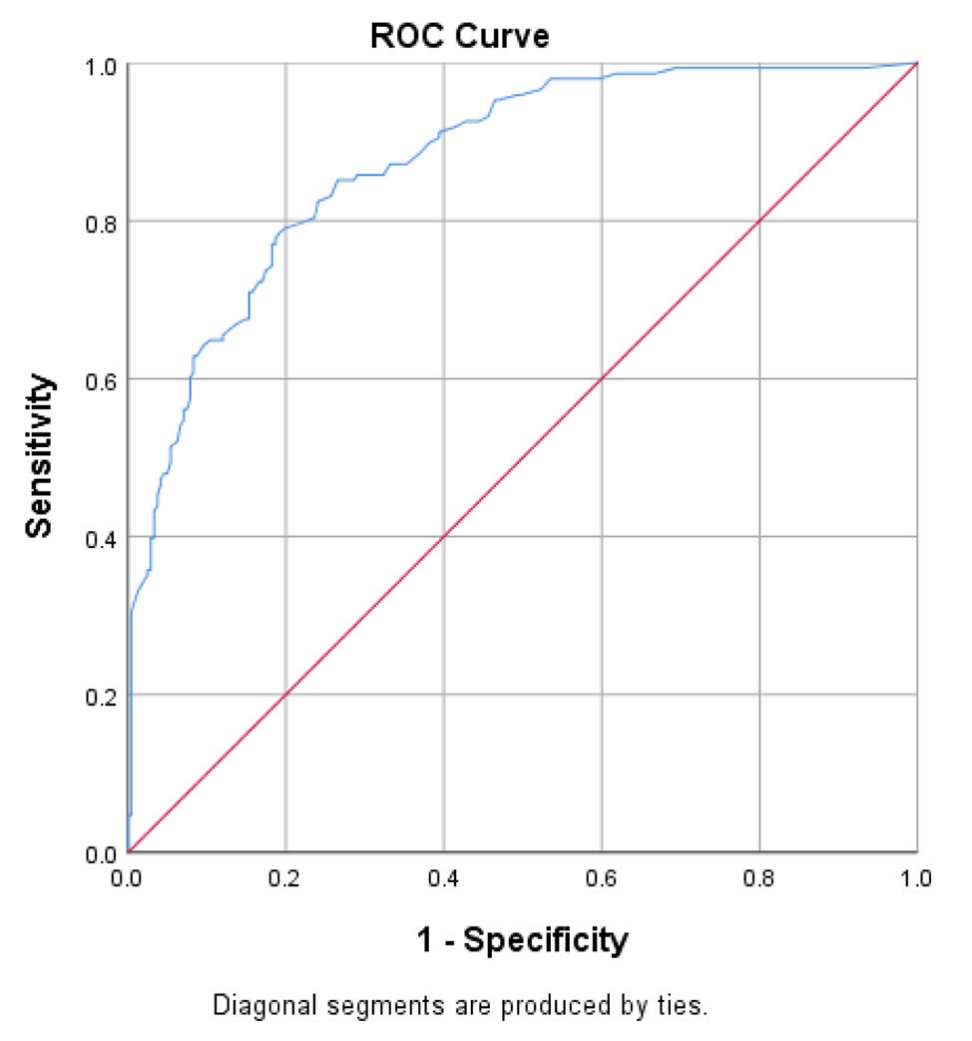
Diagnostic characteristics of the CDI total score in predicting moderate emotional distress (Emotion Thermometer cutoff score of 14). AUC = 0.875.

The CDI total score provided good overall discriminative accuracy relative to the established cutoff scores of the Emotion Thermometers (ETsum score > 14 indicates moderate emotional problems) with an AUC of 0.88 (95% CI 0.84–0.91) (see Fig. 3). The optimal cut-off score to indicate moderate emotional distress as indicated by the highest Youden Index value (0.593) was a CDI total score of 25.0 which provided a sensitivity of 78% and a specificity of 81%. A CDI total score of 37 was an optimal cut-off score for the prediction of severe emotional distress (ETsum score > 20) and provided 71% sensitivity and 86% specificity (AUC = 0.87 95% CI 0.83–0.91).

## Discussion

This study has successfully developed a comprehensive measure of cardiac-related distress. The instrument is to be known as the Cardiac Distress Inventory (CDI). Exploratory factor analysis and Rasch analysis resulted in a 55-item CDI comprising eight subscales from the items tested. Validation of the CDI against the K-6 supports its criterion validity. Evidence of concurrent validity of the total CDI score was apparent with the highest correlation obtained with the Distress Thermometer ratings and a lower correlation for the more transient COVID-19 concerns. The CDI total score provided good overall discriminative accuracy relative to the established cutoff scores of the Emotion Thermometers.

The findings demonstrate that cardiac distress is a more complex phenomenon than simply the co-occurrence of anxiety and depression. The eight subscales of the CDI assess Fear and uncertainty, Disconnection and hopelessness, Changes to roles and relationships, Overwhelm and depletion, Cognitive challenges, Physical challenges, Health system challenges, and Death concerns. Items from anxiety or depression measures comprised only a small proportion of the full item pool. We believe that these subscales comprehensively assess the broad construct of cardiac distress, thereby enhancing its clinical utility.

The instrument has broad utility for use in both research and clinical practice. In research, the full CDI can be used to provide an overall measure of distress, while individual subscales can be used to provide assessment of specific areas of distress. In clinical practice, the full inventory can be administered to assist practitioners to identify specific areas of concern for their patients or, where indicated, individual subscales could be used to assess or monitor specific areas of distress. The CDI is likely to have great utility in assisting practitioners to target and tailor therapeutic interventions to relevant areas of need for individual patients.

A particular strength of the CDI is the robust developmental process employed in this study that acknowledged both empirical evidence and clinical knowledge [39]. This has resulted in a comprehensive and fine-grained instrument. However, our search for validated instruments from which to derive items focussed largely on English-language instruments. Additionally, as Legare and colleagues have noted, studies reporting the development of instruments are generally not well-indexed in electronic databases [88] thus potentially limiting the development group’s knowledge of relevant measures.

The CDI is likely to be relevant for a range of language and cultural groups, although not in its current form. Further testing with other cultural/language groups is currently underway to develop culturally relevant versions of the instrument. This will enable cross-cultural comparison of the prevalence, severity and components of cardiac distress and will extend our understanding of the universal applicability of this construct.

### Study limitations and recommendations

The present study has a number of limitations which should be acknowledged. First, the investigation was limited by its cross-sectional research design, which did not allow assessment of the test-retest reliability and sensitivity to change of the CDI. As a result, it is premature to comment on the suitability of the CDI for use in longitudinal studies and randomised controlled trials. In order to address this, future longitudinal studies are needed to assess test-retest reliability and sensitivity to change of the CDI. Second, the study sample was restricted to those who had had an acute cardiac event, therefore its utility with those with a chronic condition such as heart failure, is unclear. A longitudinal study of heart failure patients to explore the relevance of the CDI for people experiencing distress related to a chronic condition rather than an acute cardiac event is warranted. Finally, assessment of the discriminant validity of the CDI was outside the scope of the present study. To address this, future studies could compare distress levels for specific cardiac conditions and could investigate the impact of co-morbidities on both distress prevalence and severity.

## Conclusion

In our original conceptual paper, we called for more nuanced understanding of cardiac distress and for the development of a measure that reflected its complexity. We have addressed this by developing the CDI. We look forward to the use of this 55-item version in both research studies and as a clinical assessment tool to guide counselling practice. A short-form version of the CDI will be developed for use as a screening tool in settings such as primary care, cardiac rehabilitation, and counselling services.

## Electronic supplementary material

Below is the link to the electronic supplementary material.

Supplementary Material 1: CDI FACTORS based on cluster analysis 8 FACTOR 71 items: Items sorted by factor loading

## Data Availability

Supporting data are available on request to Michael.Legrande@australianhearthealth.org.au.
