# Supplementary material for: The cardiac distress inventory: A new measure of psychosocial distress associated with an acute cardiac event
Source: BMC Cardiovasc Disord. 2022 Nov 3;22:460. doi: 10.1186/s12872-022-02897-y (PMC9633013; doi:10.1186/s12872-022-02897-y)
Supplement: Supplementary file 1 — Supplementary Material 1: CDI FACTORS based on cluster analysis 8 FACTOR 71 items: Items sorted by factor loading [file 12872_2022_2897_MOESM1_ESM.docx]

**Supplementary Table 1: CDI FACTORS BASED ON CLUSTER ANALYSIS 8 FACTOR 71 items: Items sorted by factor loading**

| Item no. | Loading | Uniqueness | Item wording |
| --- | --- | --- | --- |
| **FACTOR 1 – Fear and uncertainty 14 items** | | | |
| 18 | 1.648 | 1.458 | Thinking about having another heart event |
| 3 | 1.497 | 1.505 | Being afraid of dying |
| 2 | 1.452 | 1.626 | Thinking my condition might get worse |
| 19 | 1.415 | 1.299 | Thinking about dying |
| 39 | 1.074 | 1.570 | Dwelling on my heart condition |
| 47 | 1.046 | 1.435 | Not knowing what the future holds for me |
| 1 | 1.014 | 1.538 | Thinking I will never be the same again |
| 36 | 0.852 | 1.753 | Avoiding activities that make my heart beat faster |
| 17 | 0.844 | 1.631 | Thinking that I am not the person that I used to be |
| 5 | 0.771 | 1.803 | Not being able to cope effectively with my heart condition |
| 59 | 0.696 | 2.199 | Being unsure about how much exercise or physical activity I should be doing |
| 20 | 0.665 | 1.656 | Lacking confidence in my ability to manage my heart condition |
| 34 | 0.433 | 1.551 | Being unable to plan for the future |
| 30 | 0.352 | 1.442 | Being in places and situations that remind me of my heart event |
| **FACTOR 2 – Disconnection and hopelessness 13 items** | | | |
| 31 | 1.402 | 1.499 | Feeling lonely |
| 13 | 1.142 | 1.152 | Being unsupported by my friends or family |
| 44 | 1.141 | 0.975 | Being isolated from friends and family |
| 15 | 1.100 | 1.445 | Not having anyone to talk to who understands my difficulties |
| 43 | 1.091 | 0.924 | Being disconnected from people in my community |
| 29 | 1.029 | 0.948 | Not being supported by my friends and family in my efforts to manage my heart condition |
| 42 | 0.964 | 1.426 | Thinking my friends or family don’t understand how difficult it is living with heart disease |
| 64 | 0.881 | 1.146 | Not knowing how much I can ask for help from those around me |
| 55 | 0.782 | 1.312 | Withdrawing from people |
| 33 | 0.607 | 1.394 | Believing that others will think less of me if they know I have a heart condition |
| 53 | 0.542 | 1.406 | Being unable to accept help from others |
| 46 | 0.474 | 1.826 | Believing that others don’t have the same confidence in me as they did before my heart problem |
| 57 | 0.452 | 1.999 | Thinking that it’s my fault that I have a heart condition |
| **FACTOR 3 – Changes to roles and relationships 14 items** | | | |
| 54 | 1.082 | 1.445 | Being excluded from doing things with other people because of my heart condition |
| 69 | 1.046 | 1.486 | Being too dependent on others |
| 65 | 1.002 | 1.467 | Being unavailable to my family and friends |
| 72 | 0.948 | 1.835 | Becoming a burden to my family |
| 52 | 0.945 | 1.614 | Being unable to take care of family responsibilities |
| 71 | 0.809 | 1.560 | Thinking that my heart condition controls my life |
| 41 | 0.801 | 1.566 | Having changes in my usual roles |
| 12 | 0.687 | 2.386 | Not being able to return to work or continue working |
| 63 | 0.535 | 2.563 | Being concerned about my capacity for sexual activity |
| 74 | 0.511 | 1.650 | Thinking that my family is being overprotective of me |
| 49 | 0.500 | 1.785 | Not being able to go too far from home |
| 67 | 0.557 | 1.916 | Lacking purpose or meaning in life |
| 58 | 0.460 | 1.487 | Not knowing how my family will cope if something should happen to me |
| 68 | 0.460 | 1.289 | Not knowing what will happen to other people if I die |
| **FACTOR 4 – Overwhelm and depletion 9 items** | | | |
| 4 | 0.914 | 2.262 | Being unable to do things that I know will improve my health |
| 60 | 0.811 | 1.837 | Lacking energy |
| 51 | 0.811 | 1.601 | Being emotionally exhausted |
| 26 | 0.797 | 1.788 | Being irritated by little things |
| 48 | 0.733 | 1.707 | Not being able to sustain the lifestyle changes I need to make |
| 14 | 0.699 | 1.557 | Avoiding situations and activities |
| 35 | 0.693 | 1.769 | Having to make difficult lifestyle changes because of my heart condition |
| 21 | 0.334 | 2.091 | Being unable to deal with stress |
| 10 | 0.317 | 2.061 | Being tearful more easily than before |
| **FACTOR 5 – Cognitive challenges 4 items** | | | |
| 38 | 1.622 | 1.001 | Having difficulty remembering things |
| 50 | 1.573 | 0.830 | Forgetting things more than before |
| 9 | 1.048 | 1.354 | Having difficulty concentrating |
| 25 | 0.677 | 1.387 | Having difficulty making decisions |
| **FACTOR 6 – Physical challenges 10 items** | | | |
| 7 | 1.071 | 1.659 | Having more pain than I expected to have |
| 37 | 0.998 | 1.762 | Having chest discomfort |
| 27 | 0.705 | 1.808 | Being overly aware of my heart in my chest |
| 8 | 0.595 | 2.335 | Not sleeping well |
| 23 | 0.511 | 1.360 | Having more pain than I can deal with |
| 22 | 0.571 | 1.499 | Being physically restricted |
| 40 | 0.536 | 1.993 | Being aware that my heart is beating fast |
| 6 | 0.504 | 2.686 | Being short of breath |
| 11 | 0.481 | 1.770 | Being woken up at night by my racing heart |
| 24 | 0.330 | 2.016 | Having bad dreams or nightmares |
| **FACTOR 7 – Health system challenges 7 items** | | | |
| 66 | 1.137 | 1.220 | Not getting clear directions from my health practitioner on how to manage my heart condition |
| 56 | 0.926 | 1.410 | Not being able to get as much information as I want about my heart condition |
| 16 | 0.764 | 1.819 | Being unfamiliar with the health system |
| 70 | 0.754 | 1.303 | Not having my concerns taken seriously by my health practitioner |
| 32 | 0.700 | 1.403 | Not having access to the health care I need |
| 73 | 0.428 | 1.183 | Thinking that doctors do not believe my chest pain / discomfort is real |
| 45 | 0.418 | 1.215 | Having difficulty getting to appointments that I need to attend |

**Note:**Three items have been removed: item 62 Not accepting that this has happened to me. Coping strategy (ie denial) rather than something people are likely to find distressing; item 61 Getting lost in familiar places – did not load onto any factor and questioned the clinical relevance; item 28 Having difficulty meeting my everyday expenses – did not load highly onto any factor and conceptually did not belong in any of the factors due to it not being a consequence of cardiac distress.; Uniqueness: Gives the proportion of the common variance of the variable not associated with the factors. Uniqueness is equal to 1 – communality

**Supplemental Table 2: Cardiac Distress Inventory subscales and item-total statistics (based on n=400)**

| **Sub-scales** | **Scale Mean if Item Deleted** | **Scale Variance if Item Deleted** | **Corrected Item-Total Correlation** | **Squared Multiple Correlation** | **Cronbach's Alpha if Item Deleted** |
| --- | --- | --- | --- | --- | --- |
| **Sub-scale – Fear and uncertainty** |  |  |  |  |  |
| Thinking I will never be the same again | 5.15 | 23.41 | 0.67 | 0.49 | 0.86 |
| Not knowing what the future holds for me | 5.36 | 22.77 | 0.71 | 0.57 | 0.85 |
| Thinking that I am not the person that I used to be | 5.21 | 22.29 | 0.73 | 0.54 | 0.85 |
| Dwelling on my heart condition | 5.71 | 24.83 | 0.60 | 0.38 | 0.87 |
| Thinking my condition might get worse | 5.28 | 23.93 | 0.62 | 0.42 | 0.86 |
| Being unable to plan for the future | 5.66 | 23.93 | 0.64 | 0.50 | 0.86 |
| Avoiding activities that make my heart beat faster | 5.49 | 23.60 | 0.64 | 0.42 | 0.86 |
| Being in places and situations that remind me of my heart event | 5.94 | 26.26 | 0.49 | 0.25 | 0.88 |
| Scale mean = 6.26, Standard deviation = 5.54; standardized mean* 25.80 |  |  |  |  |  |
| **Sub-scale 2 – Disconnection and hopelessness** |  |  |  |  |  |
| Feeling lonely | 2.81 | 16.02 | 0.69 | 0.49 | 0.85 |
| Withdrawing from people | 2.91 | 16.64 | 0.69 | 0.50 | 0.85 |
| Thinking my friends or family don’t understand how difficult it is living with heart disease | 2.96 | 16.95 | 0.65 | 0.46 | 0.85 |
| Being disconnected from people in my community | 3.08 | 16.74 | 0.74 | 0.66 | 0.84 |
| Being isolated from friends and family | 3.03 | 16.45 | 0.70 | 0.62 | 0.85 |
| Believing that others don’t have the same confidence in me as they did before my heart problem | 3.00 | 18.02 | 0.50 | 0.26 | 0.87 |
| Not being supported by my friends and family in my efforts to manage my heart condition | 3.17 | 17.85 | 0.64 | 0.45 | 0.86 |
| Being unable to accept help from others | 3.12 | 18.93 | 0.44 | 0.25 | 0.87 |
| Scale mean = 3.44, Standard deviation = 4.70 ; standardized mean 14.37 |  |  |  |  |  |
| **Sub-scale 3 – Changes to roles and relationships** |  |  |  |  |  |
| Having changes in my usual roles | 5.19 | 32.85 | 0.65 | 0.45 | 0.84 |
| Thinking that my heart condition controls my life | 5.21 | 32.63 | 0.64 | 0.44 | 0.84 |
| Being unable to take care of family responsibilities | 5.30 | 33.13 | 0.63 | 0.43 | 0.84 |
| Being too dependent on others | 5.45 | 34.36 | 0.59 | 0.44 | 0.85 |
| Being unavailable to my family and friends | 5.42 | 33.70 | 0.62 | 0.40 | 0.84 |
| Becoming a burden to my family | 5.12 | 32.42 | 0.60 | 0.43 | 0.84 |
| Not being able to go too far from home | 5.30 | 33.96 | 0.54 | 0.31 | 0.85 |
| Lacking purpose or meaning in life | 5.24 | 32.94 | 0.57 | 0.36 | 0.85 |
| Not being able to return to work or continue working | 5.27 | 33.94 | 0.48 | 0.27 | 0.85 |
| Being concerned about my capacity for sexual activity | 5.26 | 34.78 | 0.42 | 0.26 | 0.86 |
| Thinking that my family is being overprotective of me | 5.55 | 37.30 | 0.34 | 0.15 | 0.86 |
| Scale mean = 5.83, Standard deviation = 6.35 ; standardized mean 17.49 |  |  |  |  |  |
| **Sub-scale 4 – Overwhelm and depletion** |  |  |  |  |  |
| Lacking energy | 4.66 | 17.96 | 0.61 | 0.45 | 0.84 |
| Being emotionally exhausted | 4.96 | 17.24 | 0.74 | 0.58 | 0.82 |
| Being irritated by little things | 5.01 | 18.68 | 0.64 | 0.42 | 0.83 |
| Avoiding situations and activities | 5.03 | 18.82 | 0.60 | 0.37 | 0.84 |
| Being unable to deal with stress | 5.13 | 18.51 | 0.61 | 0.40 | 0.84 |
| Being tearful more easily than before | 5.16 | 19.12 | 0.57 | 0.39 | 0.84 |
| Not being able to sustain the lifestyle changes I need to make | 5.33 | 19.43 | 0.55 | 0.32 | 0.84 |
| Scale mean = 5.88, Standard deviation = 4.96 ; standardized mean 27.78 |  |  |  |  |  |
| **Sub-scale 5 – Cognitive challenges** |  |  |  |  |  |
| Forgetting things more than before | 2.06 | 5.31 | 0.79 | 0.71 | 0.80 |
| Having difficulty remembering things | 1.97 | 5.17 | 0.75 | 0.70 | 0.81 |
| Having difficulty concentrating | 1.81 | 5.22 | 0.71 | 0.52 | 0.83 |
| Having difficulty making decisions | 2.18 | 5.95 | 0.62 | 0.43 | 0.86 |
| Scale mean = 2.67, Standard deviation = 3.03 ; standardized mean 22.24 |  |  |  |  |  |
| **Sub-scale 6 – Physical challenges** |  |  |  |  |  |
| Being physically restricted | 4.45 | 14.37 | 0.62 | 0.41 | 0.74 |
| Not sleeping well | 4.62 | 14.99 | 0.53 | 0.29 | 0.76 |
| Being short of breath | 4.76 | 16.23 | 0.41 | 0.22 | 0.78 |
| Being overly aware of my heart in my chest | 4.84 | 15.30 | 0.56 | 0.37 | 0.75 |
| Having chest discomfort | 4.89 | 15.59 | 0.57 | 0.37 | 0.75 |
| Having bad dreams or nightmares | 5.20 | 16.52 | 0.42 | 0.21 | 0.77 |
| Being woken up at night by my racing heart | 5.31 | 16.93 | 0.45 | 0.23 | 0.77 |
| Having more pain than I can deal with | 5.39 | 17.59 | 0.38 | 0.17 | 0.78 |
| Scale mean = 5.64, Standard deviation = 4.49 ; standardized mean 23.46 |  |  |  |  |  |
| **Sub-scale – Health system challenges** |  |  |  |  |  |
| Not getting clear directions from my health practitioner on how to manage my heart condition | 1.27 | 4.58 | 0.67 | 0.53 | 0.67 |
| Not being able to get as much information as I want about my heart condition | 1.33 | 5.00 | 0.57 | 0.41 | 0.71 |
| Not having access to the health care I need | 1.39 | 5.33 | 0.52 | 0.30 | 0.72 |
| Not having my concerns taken seriously by my health practitioner | 1.45 | 5.56 | 0.54 | 0.36 | 0.72 |
| Having difficulty getting to appointments that I need to attend | 1.48 | 6.21 | 0.37 | 0.18 | 0.77 |
| Scale mean = 1.73, Standard deviation = 2.81; standardized mean 11.47 |  |  |  |  |  |
| **Sub-scale 8 – Death concern** |  |  |  |  |  |
| Not knowing how my family will cope if something should happen to me | 2.21 | 6.40 | 0.65 | 0.58 | 0.80 |
| Being afraid of dying | 2.34 | 6.75 | 0.66 | 0.66 | 0.80 |
| Thinking about dying | 2.38 | 6.79 | 0.69 | 0.67 | 0.79 |
| Not knowing what will happen to other people if I die | 2.41 | 6.61 | 0.67 | 0.58 | 0.79 |
| Scale mean = 3.12, Standard deviation = 3.34; standardized mean 25.88 |  |  |  |  |  |

* standardized mean = mean when scale score is converted to score from 0 to 100

**Supplementary Material CDI and Scoring**


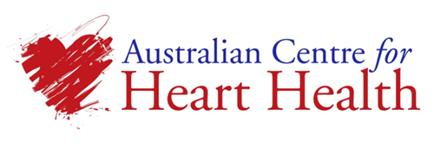


**Cardiac Distress Inventory**

Living with a heart condition can sometimes be difficult. Listed below are some issues that people living with a heart condition may experience.

Please indicate whether or not you have experienced each issue during the past four weeks by circling “Y” for yes or “N” for no. For each item that you have circled “Y”, indicate how much distress this issue has caused you **during the past four weeks** by circling, on a scale of 0 to 3, where “0” is no distress and “3” is severe distress.

| **Issue** | **Yes** | **No** | **If yes, indicate how much distress this causes for you** | | | |
| --- | --- | --- | --- | --- | --- | --- |
|  |  |  | **No distress** | **Slight distress** | **Moderate distress** | **Severe distress** |
| 1. Being physically restricted | Y | N | 0 | 1 | 2 | 3 |
| 2. Being woken up at night by my racing heart | Y | N | 0 | 1 | 2 | 3 |
| 3. Being unable to take care of family responsibilities | Y | N | 0 | 1 | 2 | 3 |
| 4. Thinking my friends or family don’t understand how difficult it is living with heart disease | Y | N | 0 | 1 | 2 | 3 |
| 5. Believing that others don’t have the same confidence in me as they did before my heart problem | Y | N | 0 | 1 | 2 | 3 |
| 6. Not getting clear directions from my health practitioner on how to manage my heart condition | Y | N | 0 | 1 | 2 | 3 |
| 7. Having more pain than I can deal with | Y | N | 0 | 1 | 2 | 3 |
| 8. Not having my concerns taken seriously by my health practitioner | Y | N | 0 | 1 | 2 | 3 |
| 9. Not be able to return to work or continue working | Y | N | 0 | 1 | 2 | 3 |
| 10. Thinking about dying | Y | N | 0 | 1 | 2 | 3 |
| 11. Thinking that I am not the person that I used to be | Y | N | 0 | 1 | 2 | 3 |
| 12. Thinking that my heart condition controls my life | Y | N | 0 | 1 | 2 | 3 |
| 13. Not knowing how my family will cope if something should happen to me | Y | N | 0 | 1 | 2 | 3 |
| 14. Being unable to accept help from others | Y | N | 0 | 1 | 2 | 3 |
| 15. Being afraid of dying | Y | N | 0 | 1 | 2 | 3 |
| 16. Thinking that my family is being overprotective of me | Y | N | 0 | 1 | 2 | 3 |
| 17. Not having access to the health care I need | Y | N | 0 | 1 | 2 | 3 |
| 18. Not being able to go too far from home | Y | N | 0 | 1 | 2 | 3 |
| 19. Having chest discomfort | Y | N | 0 | 1 | 2 | 3 |
| 20. Being irritated by little things | Y | N | 0 | 1 | 2 | 3 |
| 21. Forgetting things more than before | Y | N | 0 | 1 | 2 | 3 |
| 22. Having difficulty getting to appointments that I need to attend | Y | N | 0 | 1 | 2 | 3 |
| 23. Having difficulty making decisions | Y | N | 0 | 1 | 2 | 3 |
| 24. Being overly aware of my heart in my chest | Y | N | 0 | 1 | 2 | 3 |
| 25. Being isolated from friends and family | Y | N | 0 | 1 | 2 | 3 |
| 26. Having difficulty concentrating | Y | N | 0 | 1 | 2 | 3 |
| 27. Not being able to sustain the lifestyle changes I need to make | Y | N | 0 | 1 | 2 | 3 |
| 28. Thinking I will never be the same again | Y | N | 0 | 1 | 2 | 3 |
| 29. Being tearful more easily than before | Y | N | 0 | 1 | 2 | 3 |
| 30. Not being supported by my friends and family in my efforts to manage my heart condition | Y | N | 0 | 1 | 2 | 3 |
| 31. Feeling lonely | Y | N | 0 | 1 | 2 | 3 |
| 32. Thinking my condition might get worse | Y | N | 0 | 1 | 2 | 3 |
| 33. Avoiding activities that make my heart beat faster | Y | N | 0 | 1 | 2 | 3 |
| 34. Being unable to deal with stress | Y | N | 0 | 1 | 2 | 3 |
| 35. Becoming a burden to my family | Y | N | 0 | 1 | 2 | 3 |
| 36. Not knowing what the future holds for me | Y | N | 0 | 1 | 2 | 3 |
| 37. Lacking energy | Y | N | 0 | 1 | 2 | 3 |
| 38. Being in places and situations that remind me of my heart event | Y | N | 0 | 1 | 2 | 3 |
| 39. Having changes in my usual roles | Y | N | 0 | 1 | 2 | 3 |
| 40. Not sleeping well | Y | N | 0 | 1 | 2 | 3 |
| 41. Not being able to get as much information as I want about my heart condition | Y | N | 0 | 1 | 2 | 3 |
| 42. Being disconnected from people in my community | Y | N | 0 | 1 | 2 | 3 |
| 43. Being unable to plan for the future | Y | N | 0 | 1 | 2 | 3 |
| 44. Being concerned about my capacity for sexual activity | Y | N | 0 | 1 | 2 | 3 |
| 45. Avoiding situations and activities | Y | N | 0 | 1 | 2 | 3 |
| 46. Withdrawing from people | Y | N | 0 | 1 | 2 | 3 |
| 47. Dwelling on my heart condition | Y | N | 0 | 1 | 2 | 3 |
| 48. Being unavailable to my family and friends | Y | N | 0 | 1 | 2 | 3 |
| 49. Being too dependent on others | Y | N | 0 | 1 | 2 | 3 |
| 50. Having bad dreams or nightmares | Y | N | 0 | 1 | 2 | 3 |
| 51. Having difficulty remembering things | Y | N | 0 | 1 | 2 | 3 |
| 52. Being short of breath | Y | N | 0 | 1 | 2 | 3 |
| 53. Not knowing what will happen to other people if I die | Y | N | 0 | 1 | 2 | 3 |
| 54. Being emotionally exhausted | Y | N | 0 | 1 | 2 | 3 |
| 55. Lacking purpose or meaning in life | Y | N | 0 | 1 | 2 | 3 |

**SCORING OF THE CARDIAC DISTRESS INVENTORY**

The Cardiac Distress Inventory (CDI) is a 55-item measure comprised of eight factors: Fear and uncertainty (8 items); Disconnection and hopelessness (8 items); Changes to roles and relationships (11 items); Overwhelm and depletion (7 items); Cognitive challenges (4 items); Physical challenges (8 items); Health system challenges (5 items); Death concern (4 items).

All items are scored 0 to 3

0 - item not endorsed OR item endorsed but no distress level indicated
1 - item endorsed and low level of distress indicated
2 - item endorsed and medium level of distress indicated
3 - item endorsed and high level of distress indicated

**Fear and uncertainty 8 items:** 11, 28, 32, 33, 36, 38, 43, 47

**Disconnection and hopelessness 8 items:** 04, 05, 14, 25, 30, 31, 46, 42

**Changes to roles and relationships 11 items;** 03, 09 ,12, 16, 18, 35, 39 ,44, 48, 49, 55

**Overwhelm and depletion 7 items:** 20, 27, 29, 34 37, 45, 54

**Cognitive challenges 4 items:** 21 ,23 26. 51

**Physical challenges 8 items:** 01, 02, 07, 19, 24, 40, 50, 52

**Health system challenges 5 items:** 06, 08, 17, 22, 41

**Death concern 4 items:** 10, 13, 15, 53

**TOTAL CDI SCORE 55 items:** Sum of all 55 item

**SPSS Syntax**

**Fear and uncertainty 8 items**

Sum of items: 11, 28, 32, 33, 36, 38, 43, 47
Get standard score out of 100 by dividing total sum/total possible score (24) x 100

Example SPSS syntax: this sums the 8 items and provides mean substitution for missing. In these examples we have put the prefix CDS (scale no)_ before all item numbers.

COMPUTE CDS1= MEAN.4 (CDS1_11, CDS1_28, CDS1_32, CDS1_38, CDS1_43, CDS1_33, CDS1_47, CDS1_36) * 8.
EXECUTE.

* Calculate standard score out of 100.

COMPUTE CDS1_100 = CDS/24 * 100.
Execute.

**Disconnection and hopelessness 8 items**

Sum of items: 04, 05, 14, 25, 30, 31, 46, 42
Get standard score out of 100 by dividing total sum/total possible score (24) x 100

Example SPSS syntax: this sums the 8 items and provides mean substitution for missing

COMPUTE CDS2= MEAN.4 (CDS2_31, CDS2_46, CDS2_04, CDS2_42, CDS2_25, CDS2_05, CDS2_30, CDS2_14) * 8.
EXECUTE.

* Calculate standard score out of 100.

COMPUTE CDS2_100 = CDS2/24 * 100.
Execute.

**Changes to roles and relationships 11 items**

Sum of items 03, 09 ,12, 16, 18, 35, 39 ,44, 48, 49, 55
Get standard score out of 100 by dividing total sum/total possible score (33) x 100

Example SPSS syntax: this sums the 11 items and provides mean substitution for missing

COMPUTE CDS3= MEAN.6 (CDS3_09, CDS3_39, CDS3_18, CDS3_03, CDS3_44, CDS3_48, CDS3_55, CDS3_49, CDS3_12, CDS3_35, CDS3_16) * 11.
EXECUTE.

* Calculate standard score out of 100.

COMPUTE CDS3_100 = CDS3/33 * 100.
Execute.

**Overwhelm and depletion 7 items**

Sum of items 20, 27, 29, 34 37, 45, 54
Get standard score out of 100 by dividing total sum/total possible score (21) x 100

COMPUTE CDS4= MEAN.4 (CDS4_29, CDS4_45, CDS4_34, CDS4_20, CDS4_27, CDS4_54, CDS4_37) * 7.
EXECUTE.
COMPUTE CDS4_100 = CDS4/21 * 100.
Execute

**Cognitive challenges 4 items**

Sum of items, 21 ,23 26. 51
Get standard score out of 100 by dividing total sum/total possible score (12) x 100

COMPUTE CDS5= MEAN.2 (CDS5_23, CDS5_51, CDS5_21, CDS5_26) * 4.
EXECUTE.
COMPUTE CDS5_100 = CDS5/12 * 100.
Execute.

**Physical challenges 8 items**

Sum of items 01, 02, 07, 19, 24, 40, 50, 52
Get standard score out of 100 by dividing total sum/total possible score (24) x 100

COMPUTE CDS6= MEAN.4 (CDS6_02, CDS6_01, CDS6_07, CDS6_50, CDS6_24, CDS6_19, CDS6_52, CDS6_40) * 8.
EXECUTE.

COMPUTE CDS6_100 = CDS6/24 * 100.
Execute.

**Health system challenges 5 items**

Sum of items, 06, 08, 17, 22, 41
Get standard score out of 100 by dividing total sum/total possible score (15) x 100

COMPUTE CDS7= MEAN.3 (CDS7_17, CDS7_22, CDS7_41, CDS7_06, CDS7_08) * 5.
EXECUTE.

COMPUTE CDS7_100 = CDS7/15 * 100.
Execute.

**Death concern 4 items**

Sum of items 10, 13, 15, 53
Get standard score out of 100 by dividing total sum/total possible score (12) x 100

COMPUTE CDS8= MEAN.2 (CDS8_15, CDS8_10, CDS8_13, CDS8_53) * 4

COMPUTE CDS8_100 = CDS8/12 * 100.
Execute.

**TOTAL CDI SCORE 55 items**

Sum of all 55 items
Get standard score out of 100 by dividing total sum/total possible score (165) x 100
For missing items substitute mean value of present items providing at least half of the scale (28 items) has been answered.

SPSS syntax:
COMPUTE CDSTOT= MEAN.28 (CDS1_11 , CDS1_28 , CDS1_32 , CDS1_38 , CDS1_43 , CDS1_33 , CDS1_47 , CDS1_36, CDS2_31 ,CDS2_46 , CDS2_04 , CDS2_42 , CDS2_25 , CDS2_05 , CDS2_30 , CDS2_14, CDS3_09 ,CDS3_39 , CDS3_18 , CDS3_03 , CDS3_44 , CDS3_48 , CDS3_55 , CDS3_49 , CDS3_12 , CDS3_35, CDS3_16, CDS4_29 ,CDS4_45 , CDS4_34 , CDS4_20 , CDS4_27 , CDS4_54 , CDS4_37,CDS5_23 ,CDS5_51 , CDS5_21 , CDS5_26, CDS6_02 ,CDS6_01 , CDS6_07 , CDS6_50 , CDS6_24 , CDS6_19 , CDS6_52 , CDS6_40, CDS7_17 ,CDS7_22 , CDS7_41 , CDS7_06 , CDS7_08, CDS8_15 ,CDS8_10 , CDS8_13 , CDS8_53) * 55.
EXECUTE.

COMPUTE CDSTOTAL100= CDSTOT/165 * 100.
Execute.
